# Multi-drug resistant gram negative infections and use of intravenous polymyxin B in critically ill children of developing country: retrospective cohort study

**DOI:** 10.1186/s12879-014-0626-9

**Published:** 2014-11-28

**Authors:** Naveed-ur-Rehman Siddiqui, Farah Naz Qamar, Humaira Jurair, Anwarul Haque

**Affiliations:** Department of Pediatrics and child Health, Aga Khan University, Stadium Road, Karachi, 74800 Pakistan

**Keywords:** Pediatric intensive care unit (PICU), Multi-drug resistance (MDR), Gram negative organism, Polymyxin B

## Abstract

**Background:**

Patients in pediatric intensive care Units (PICU) are susceptible to infections with antibiotic resistant organisms which increase the morbidity, mortality and cost of care.

To describe the clinical characteristics and mortality in patients with Multi-Drug Resistant (MDR) gram negative organisms. We also report safety of Polymyxin B use in these patients.

**Methods:**

Files of patients admitted in PICU of Aga Khan University Hospital, from January 2010 to December 2011, one month to 15 years of age were reviewed. Demographic and clinical features of patients with MDR gram negative infections, antibiotic susceptibility pattern of isolates, discharge disposition and adverse effects of Polymyxin B were recorded.

**Results:**

A total of 44.8/1000(36/803) admitted patients developed MDR gram negative infections, of which 47.2%(17/36) were male, with mean age of 3.4 yrs(+/−4.16). *Acinetobacter Species* (25.5%) was the most frequently isolated MDR organisms followed by *Klebsiella Pneumoniae* (17%). Sensitivity of isolates was 100% to Polymyxin B, followed by Imipenem (50%), and piperacillin/tazobactem (45%). The crude mortality rate of patients with MDR gram negative infections was 44.4% (16/36). Fourteen of 36 patients received Polymyxin B and 57.1%; (8/14) of them were cured. Nephrotoxicity was observed in 21.4% (3/14) cases, none of the patients showed signs of neuropathy.

**Conclusion:**

Our study highlights high rates of Carbapenem resistant gram negative isolates, leading to increasing use of Polymyxin B as the only drug to combat against these critically ill children. Therefore, we emphasizeon Stewardship of Antibiotics and continuous surveillance system as strategies in overall management of these critically ill children.

## Background

The emergence of bacterial resistance is a threat particularly in Intensive Care Units (ICUs) settings, where these organisms are prevalent and their treatment becomes a challenge [1]. Multi-Drug Resistant (MDR) *Staphylococcus Aureus* particularly ------ Methicillin Resistant Staphylococcus Aureus (MRSA) has been in the limelight for long and has been controlled in high income countries to some extent by stringent infections control measures. However, less attention has been given to gram negative infections until recently, when antimicrobial resistance among gram negative organisms and their spread raised serious public health concerns [2]-[4].

High rates of antibiotic resistant pathogens have been reported globally, in particular there are reports of rising trends from low and low middle income countries (LMIC) [4]. The most concerning fact is the development and spread of Klebsiella Pneumoniae Carbapenemase(KPC), Carbapenemase Resistant Enterobacteriacea (CRE) which leave very few antibiotic choices. Rates of resistance are much higher in critically ill patients. Resistance to Imipenem for bloodstream isolates of *Acinetobacter Baumannii* from ICU patients has been reported to be as high as 85% [5]. Similarly, high resistance rates for P*. Auregunosa* isolates to Imipenem and quinolones have been reported [6],[7]. gram negative pathogens that are resistant to polymyxin are also being reported from many countries and are a cause of mortality and high health care costs [8]-[10]. The reasons for development of resistance are numerous and related to antibiotic overuse and misuse in conjunction with poor infection control practices in health care settings [11]. The development of resistance to almost all classes of antibiotics is an alarming situation leaving physicians with only few treatment options [1]. Development of new antibiotics is limited and can be realized from the fact that only 5 new antibiotics have been developed and approved for use in the last decade [3]. Consequently there has been a resurgence of old antibiotics such as polymyxin as last resort for the treatment of infections caused by MDR gram negative pathogens [12]-[15].

The rising trend of highly resistant gram negative infections has also been described in our country [16]-[18]. 94% and 75% of the total gram negative isolates were resistant to one or more antibiotics at the level of 10ug/ml and 30ug/ml respectively [17]. But these studies were done in adults and neonatal age group [16]-[18]. As there is lack of data among pediatric age group especially in critically ill children, so we aim to describe the clinical characteristics, antibiotic sensitivity pattern and mortality in patients with Multi-Drug Resistant (MDR) gram negative organisms, and also report the safety of polymyxin B use among critically ill pediatric patients.

## Methods

Files of all patients admitted in the PICU of Aga Khan University Hospital, from January 2010 to December 2011, aged one month to 15 yrs were reviewed. The study was approved by Ethical Review Committee of Aga Khan University Hospital for the review of patients medical record. (2736-PED-ERC-13) As per policy of PICU, blood and urine cultures of all cases were sent at the time of admission to PICU. CSF cultures were sent for all sick infants <3 months of age and in older children if signs and symptoms of meningitis were present. Tracheal cultures were sent by tracheal aspiration for all ventilated patients after 48 hours of intubation or earlier if clinical signs are suggestive of chest infection were present.

All cultures were performed in the clinical laboratory of Aga Khan University Hospital using standard laboratory methods and susceptibility was performed according to CLSI guidelines [19]. For blood cultures, Bactec 9240 blood culture instrument was used. gram staining was performed on positive cultures in order to identify the organisms likely to be present. After identification conventional biochemical tests were done to identify the specific gram negative organism. If not identified, organisms were then identified on analytical profile index (ANI) on BioMerieux. For Urinary tract infections (UTIs), all specimens were inoculated on cysteine lactose Electrolyte Deficient (CLED) Agar. If identified any growth > 10^5^ CFU/ml, was identified, gram staining was done, followed by conventional biochemical tests to identify the specific gram negative organisms. For respiratory secretions (tracheal & bronchoalveolar lavage), all specimens were inoculated for quantitative culture, if identified 10^5^ – 10^4^ CFU/ml, then gram staining was done followed by conventional biochemical test to identify specific gram negative organisms. For wound cultures, specimens (swabs/tissues/pus) were inoculated for semiquantitative cultures. If growth occured, gram staining was done, followed by conventional biochemical tests to identify the specific gram negative organisms. Antibiotic susceptibility testing was done by Kirby-Bener’s disk diffusion method and E test for all the isolated bacteria on Mueller Hinton Agar (MHA) to determine their resistance pattern against common antibiotics following standards of clinical laboratory standard institute (CLSI, formerly National Committee for Clinical Laboratory Standards [NCCLS]) [19],[20]. Multidrug resistant organisms with resistance to imepenem were tested for sensitivity to polymyxin B. A colony count of 10^5^ in tracheal culture was considered significant [21]. Culture proven sepsis was defined as one or more positive blood culture collected during stay in the PICU. Diagnosis of coagulase-negative staphylococcal sepsis required at least 2 positive blood cultures [22]. For each patient, positive cultures from each site were counted as separate events. All information was recorded on a structured proforma. The information collected included demographic characteristics of patients, site of infection, antibiotic sensitivity of the isolate and the mortality rate among the infected patients. MDR was defined as resistance to 3 or more antimicrobial groups against which it has been tested [23]. Final disposition was defined as death related to infection (death in the setting of clinical evidence of active infection or within 5 days after the last positive culture result), death unrelated to infection (death after an episode of infection but due to causes independent of the infectious process), or survival. Mortality was calculated by dividing the number of patients who died within 5 days of an infection with MDR-gram negative infections by the total number of subjects with MDR-gram negative infection. Data on polymyxin B use among these patients, (dose, prior and concomitant antibiotic use and, development of nephrotoxicity) was also recorded. Nephrotoxicity was assessed by the measurement of serum creatinine levels before and after the use of polymyxin B, and an increase of 100% serum creatinine level from the baseline was considered as a marker for nephrotoxicity. Evaluation of neurotoxicity was analyzed by going through the recorded information on neurological examination before and after the use of polymyxin B. The development of neurological signs after the start of polymyxin in the absence of any other precipitating cause was taken as a marker for neurotoxicity due to polymyxin B. The mortality rate amongst patients in whom polymyxin B was used, was also reported.

## Results

A total of 803 patients were admitted in the PICU during the study period, of which 44.8/1000 (36/803) admitted patients developed 47 episodes of MDR-gram negative infections. Most (47.2%, 17/36) were male, 52.7% (19/36) being under 1 year of age with mean age of 3.4 years (+/− 4.16). The clinical and demographic characteristics of patients who developed MDR gram negative infections are shown in Table 1. Majority of the organisms were isolated from respiratory tract 59.6% (28/47) followed by bloodstream infection (BSI) 25.5% (12/47), with *Acinetobacter Species* (25.5%) being the most frequently isolated organism followed by *Klebsiella Pneumoniae* (17%), *Escherichia coli* (14.9%) and *Pseudomonas Aeruginosa* 12.8% as in Table 2. The mortality rate among MDR-gram negative infective critically ill pediatric patients was 44.5% (16/36), 75% were under 1 year of age, as compared to overall mortality of 12.3%(99/803) in our unit. Isolates of *Acinetobacter Species* were resistant to all antibiotics including carbapenems and tazobactems and were sensitive only to polymyxin B (100%) and tobramycin (66.6%). Other organisms also showed variable degree of resistance to different classes of antibiotics, the frequency of sensitivities to the various groups of antibiotics are shown in Table 3. The characteristic of patients in whom polymyxin was used is shown in Table 4. Nephrotoxicity was observed in 21.4% (3/14) patients, of them all died, none of the subjects developed clinical signs of neurotoxicity. The mortality rate among patients in whom Polymyxin B was used was 42.9% (6/14).

**Table 1:** **Clinical characteristics of critically ill pediatric patients with MDR-Gram negative infections**

Characteristics	n = 36 n (%)
Height in cms (Mean+/− SD)	79.55(+/−31.2)
Weight in kg (Mean+/− SD)	11.7(+/−11.4)
Ages in yrs. (Mean +/− SD)	3.4(+/−4.16)
<1 year	19(52.7%)
1-5 years	7(19.4%)
>5 years	10(27.8%)
Gender	
Male	17(47.2%)
System Involved	
CVS	14(38.9%)
CNS	7(19.4%)
Respiratory	6(16.7%)
MOD	3(8.3%)
Hematological Malignancies	2(5.6%)
Renal	1(2.8%)
GIT	1(2.8%)
Others	2(5.6%)
Outcome	
Discharged	20(55.6%)
Expired	16(44.4%)

MDR: Multi-Drug resistant, CVS: Cardiovascular system, CNS: Central nervous system, MOD: Multi-organ dysfunction, GIT: Gastrointestinal tract.

**Table 2:** **Pathogens isolated with site of infections (n = 47)**

Organisms	n (%)	Blood	Respiratory	Urine	Wound	Blood + Respiratory
		n (%)	n (%)	n(%)	n (%)	n (%)
***Acinetobacter Species***	12(25.5%)	2	8	0	0	2
***Klebsiella Pneumoniae***	8(17.0%)	0	7	0	0	1
***Pseudomonas Aeruginosa***	6(12.8%)	2	4	0	0	0
***Escherichia Coli***	7(14.9%)	3	2	2	0	0
***Enterobacter Species***	5(10.6%)	2	3	0	0	0
***Stenotrophomonas Maltophilia***	4(8.5%)	0	3	0	0	1
***Burkholderia Cepacia***	2(4.3%)	2	0	0	0	0
***Pseudomonas Species***	3(6.4%)	1	1	0	1	0
**Total n (%)**	47(100%)	12(25.5%)	28(59.6%)	2(4.3%)	1(2.1%)	4(8.5%)

**Table 3:** **Antibiotic sensitivity of the multi-drug resistant gram negative isolates**

Organisms	Pip/Taz	Imepenem	Polymyxin B	Gentamycin	Amikacin	Aztreonam	Ceftriaxone	Ceftazidime	Ofloxacin	Cefuroxime	Amp/clav	Tobramycin
*Acinetobacter Species*	0/12	0/12	12/12	0/12	0/12	0/6	0/12	0/12	0/12	0/8	0/2	6/9
	(0%)	(0%)	(100%)	(0%)	(0%)	(0%)	(0%)	(0%)	(0%)	(0%)	(0%)	(66.6%)
*Klebsiella Pneumoniae*	5/8	6/8	3/3	0/8	3/8	0/8	0/8	0/2	2/8	0/7	2/7	
	(62.5%)	(75%)	(100%)	(0%)	(37.5%)	(0%)	(0%)	(0%)	(25%)	(0%)	(28.6%)	
*Pseudomonas Aeruginosa*	3/5	1/6	5/5	1/6	2/6	¼	0/3	2/4	1/6	0/2	--	0/1
	(60%)	(16.7%)	(100%)	(16.7%)	(33.3%)	(25%)	(0%)	(50%)	(16.7%)	(0%)		(0%)
*Escherichia Coli*	5/7	7/7	--	5/7	7/7	0/7	0/7	0/1	4/7	0/6	3/7	--
	(71.5%)	(100%)		(71.5%)	(100%)	(0%)	(0%)	(0%)	(57.2%)	(0%)	(42.8%)	
*Enterobacter Species*	¾	4/4	--	1/5	1/5	0/4	0/4	0/1	¼	0/4	¼	--
	(75%)	(100%)		(20%)	(20%)	(0%)	(0%)	(0%)	(25%)	(0%)	(25%)	
*Stenotrophomonas Maltophilia*	½	--	--	0/1	0/1	0/2	0/2	½	¾	--	--	--
	(50%)			(0%)	(0%)	(0%)	(0%)	(50%)	(75%)			
*Burkholderia Cepacia*	--	½	--	--	--	--	--	2/2	2/2	--	--	--
		(50%)						(100%)	(100%)			
*Pseudomonas Species*	½	2/3	3/3	1/3	1/3	0/3	0/3	0/1	1/3	0/1	--	0/1
	(50%)	(66.6%)	(100%)	(33.3%)	(33.3%)	(0%)	(0%)	(0%)	(33.3%)	(0%)		(0%)
n (%)	18/40	21/42	23/23	8/42	14/42	1/34	0/39	5/25	14/46	0/28	6/20	6/11
	(45%)	(50%)	(100%)	(19.1%)	(35.8%)	(3%)	(0%)	(40%)	(30.5%)	(0%)	(30%)	(54.6%)

Pip/Taz = Piperacillin/Tazobactem Amp/Clav = Ampicillin + Clavulanic acid.

**Table 4:** **Characteristics of pediatric critically ill patients with MDR-Gram negative organism with Intravenous polymyxin B use**

Characteristics	n = 14 n(%)
**Age median (range)**	**7 month ( 1 month - 12 yrs)**
**Gender**	
Males	7(50%)
**Underlying disease**	
CVS	8(57.1%)
CNS	4(28.6%
Respiratory	2(14.3%)
**Site of infection**	
Respiratory	11(78.6%)
BSI	1(7.1%)
BSI + Respiratory	2(14.3%)
**Isolated organisms in Blood (n = 3)**	
*Acinetobacter Species*	1
*Pseudomonas Aeruginosa*	1
*Klebsiella Pneumoniae*	1
**Isolated organisms in respiratory secretions (n = 15)**	
*Acinetobacter Species*	9
*Pseudomonas Aeruginosa*	4
*Klebsiella Pneumoniae*	1
*Enterobacter species*	1
**Antibiotics used prior to Polymyxin B**	Ceftriaxone, Meropenem, Vancomycin
**Antibiotics used concomitantly with Polymyxin B**	Meropenem, Vancomycin, Tobramycin, Ciprofloxacin
**PICU stay before instillation of IV Polymyxin B in days** median (range)	7.0(1–17 days)
**Duration of IV Polymyxin B treatment in days** median (range)	8.5(2–14 days)
**Dosage of Polymyxin B**	40,000 IU/kg/day
**Nephrotoxicity**	3(21.4%)
**Serum creatinine before Polymyxin B use** (mg/dl) median (range)	0.41(0.1-1.2)
**Serum creatinine after Polymyxin B use** (mg/dl) median (range)	0.55(0.1-1.6)
**Neurotoxicity**	0
**Outcome**	
Expired	6(42.9%)
Survived	8(57.1%)

## Discussion

We report a high incidence of MDR gram negative infections. In another cohort the cumulative incidence of gram negative blood stream infection was 5.4/1000 hospital admissions of which 39% were MDR gram negative isolates [24]. Another study in ours institute showed comparable incidence of 55/1000 of late onset *Klebsiella Pneumoniae* sepsis, of which 80% were resistant to multiple antibiotics, but this study involved only neonates [16]. Most of our patients were infants constituting 52%, which is comparable to another study with >50% patient sunder 2 years of age excluding the neonates [24].

Among the MDR-gram negative rods, Acinetobacter *species* followed by *Klebsiella Pneumoniae*, *Escherichia coli*, and *Pseudomonas Aeruginosa* were the most frequently isolated pathogens as reported earlier [25]. However in, another study Klebsiella Pneumonia and Psuedomonas were the most common MDR isolates [24]. *Acinetobacter species* that are drug resistant and hence difficult to treat are increasingly being reported [26]-[28]. Carbapenem resistant Klebsiella and pseudomonas are also on the rise. One study had shown an increasing trend of *Klebsiella Pneumoniae* resistance to Amikacin, Flouroquinolones, Piperacillin/Tazobactems, and Imipenem among neonates [16]. Another study in adults showed 94% of resistance at the level of 10ug/ml and 75% of resistance at the level of 30ul/ml among clinically isolated gram negative organisms [17]. According to a recent report from Centre for Disease Control and prevention (CDC), more than 70% of bacteria responsible for health-care associated infections (HAIs) are resistant to at least one of the antibiotics commonly selected to treat them [1]. Therefore, increasing use of broad spectrum antibiotics and lack of good stewardship has contributed to these MDR infections [29]. With limited antibiotic options, Implementation of good antibiotic stewardship program, Continuous surveillance System, and stringent infection control seem to be the best available option.

We report a high mortality rate of 42.9% in subjects with MDR gram negative infections of which 75% were less than 1 year of age as compared to the overall mortality rate of 12.3% in our unit. Earlier studies have reported an overall mortality between 10–53.6% [24],[30]-[33]. However it is difficult to ascertain whether resistance was the cause of death or other correlates like severity of the underlying illness, prolonged antibiotic treatment and comorbidities played a role. The high rates of infection among critically ill patients are related to exposure to invasive procedures, underlying illness, prolonged ICU stay, antibiotic exposure and patient to patient spread [34].

With the exception of Tigecycline, a relatively recently approved antibiotic active against MDR *Acinetobacter Baumannii*, [35] no new antibiotic against the MDR-gram negative bacteria is even in the drug development pipeline [36]. polymyxin B and colistin (Polymyxin E) are the two polymyxins used clinically; but the knowledge on pharmacokinetics and pharmacodynamics is extremely limited, [37] and most was obtained before 1980s [12],[15].

There are limited reports on use of polymyxin B in pediatric age group [30],[31]. There were only few studies investigating the use of polymyxin B for the treatment of infections caused by MDR-gram negative pathogens, mostly Acinetobacter and Pseudomonas [38]-[40]. We report data of 14 critically ill pediatric patients, who received intravenous polymyxin B but despite this almost half of them expired. Previously reported mortality rate among MDR-gram negative infective patients with Polymyxin use was 20-54% [38]-[41] which is comparable to our data. However it is tough to decide whether the Polymyxin B was the cause of death or other risk factors like severity of the underlying disease and comorbidities etc. played a role. Nephrotoxicity and neurotoxicity were the most potential toxicities with parenteral administration of Polymyxin B [15],[42]. Incidence of nephrotoxicity ranges from 0-37% [42]. We observed a similar rate of nephrotoxicity. It is difficult to ascertain whether this was due to progression of the acute kidney injury due to underlying disease or due to the use of other nephrotoxic drug concomitantly used with Polymyxin or primarily an adverse effect of Polymyxin B itself. Neurotoxicity due to Polymyxin B is usually mild and resolves after discontinuation of therapy [42]. Signs of Polymyxin B induced neurotoxicity include dizziness, generalized muscle weakness, facial or peripheral paresthesia, partial deafness, visual disturbances, vertigo, confusion, hallucinations, seizures, ataxia, and neuromuscular weakness [39]. However, we did not find any neurotoxicity associated with the use of intravenous Polymyxin B.

The limitations of this study are a retrospective collection of data, limited sample size limiting determination of risk factors for mortality among MDR gram negative patients and inability to comment on the nephrotoxicity of polymyxin.

## Conclusion

Our study highlights high rates of carbapenem resistant gram negative isolates, leading to increasing use of polymyxin B as only drug to combat against these critically ill children. Therefore, we stress on increasing stewardship of antibiotics and continuous surveillance system as strategies in overall management of these critically ill children.
